# Enantiomer sensing enables social avoidance by bacterial spores

**DOI:** 10.1016/j.isci.2026.114967

**Published:** 2026-02-10

**Authors:** Colin J. Comerci, Todd Kwang-Tao Chou, Ramina Amino, Maja Bialecka, Jordi Garcia-Ojalvo, Gürol M. Süel

**Affiliations:** 1 Department of Molecular Biology, School of Biological Sciences, University of California, San Diego, La Jolla, CA 92093, USA; 2 Department of Medicine and Life Sciences, Universitat Pompeu Fabra, Barcelona 08003, Spain; 3 Center for Bioengineering, University of California, San Diego, La Jolla, CA 92093, USA; 4 Center for Microbiome Innovation, University of California, San Diego, La Jolla, CA 92093, USA

**Keywords:** Microbiology

## Abstract

The role of molecular chirality in shaping interactions among organisms remains unclear. We discovered that amino acid stereochemistry provides a social avoidance mechanism for *Bacillus subtilis* spores. Germination of spores is triggered by L-alanine and competitively inhibited by its enantiomer D-alanine, produced almost exclusively by bacteria. The biological role of this enantiomer sensing remains unclear. We quantified the L- and D-alanine concentrations secreted by over 20 diverse bacterial species. We find that enantiomer ratios secreted by these species are located just beyond the germination response range for *B. subtilis* spores. Spores thus avoid germination when sensing other species. By forcing germination in pairwise co-cultures, we show that the presence of another bacterial species is often detrimental to germinating spores. Spores thus appear to exploit the L-/D-alanine ratio as a social avoidance mechanism, revealing a benefit for enantiomer sensing and potential importance of amino acid handedness in ecosystems.

## Highlights

• Bacterial spores sense the alanine enantiomer ratio and decide when to germinate
• Most bacterial species produce enantiomer ratios that keep spores dormant
• Dormant spores have a competitive advantage when in contact with other species
• Spores thus use alanine enantiomer sensing as a social avoidance mechanism

## Graphical abstract

**Figure:**
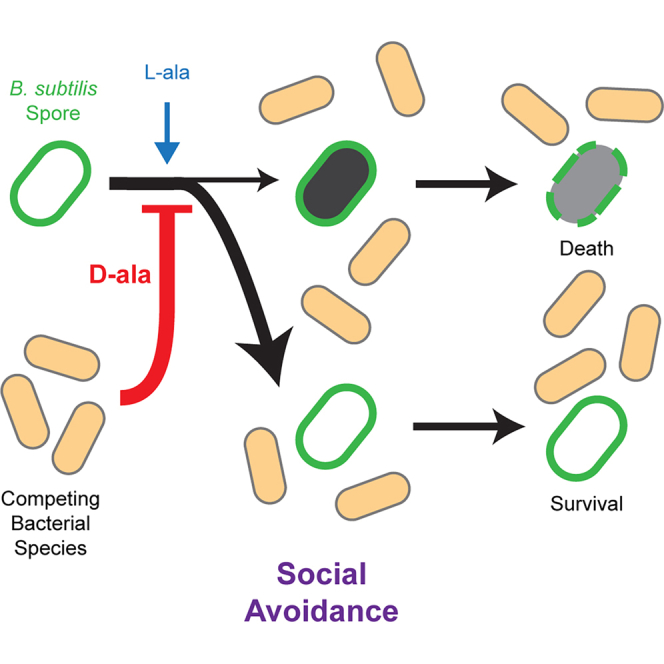

## Introduction

Amino acids are most widely known for their role as the structural building blocks of proteins. They are chiral molecules that can exist in two distinct orientations (handedness), called the L- and D-enantiomers. Many enzymes, including ribosomes,^1^ are highly stereospecific, and the vast majority of cells use only L-enantiomers. In fact, life on planet Earth has a marked preference for L-amino acids.^2^ Bacteria, however, utilize D-enantiomers (primarily D-alanine and D-glutamate) to build the peptidoglycan layer of their cell walls.^3^^,^^4^ The abundance of bacteria around the globe leads to the presence of D-amino acids in many environments, including the gut,^5^^,^^6^^,^^7^ soil,^8^ water,^9^^,^^10^^,^^11^^,^^12^ and many foods.^13^ The biological impact and ecological roles that D-amino acids may play in the environment remain an active area of investigation.

In addition to their structural role in proteins, amino acids can also act as signaling molecules. Specifically, while L-amino acids typically regulate metabolic pathways,^14^ D-amino acids can act as signals in a variety of contexts,^15^^,^^16^^,^^17^ ranging from regulating signaling in the cerebral cortex^18^ to bacterial cell-wall remodeling.^19^ One important area where amino acids act as signals is bacterial spore germination. Under starvation or stress, some species of bacteria can form dormant spores.^20^ This dormant cellular state has little to no metabolic activity, is resistant to stress, and can remain in this inactive state for years.^21^ It is widely accepted that spores germinate when presented with specific nutrient signals to resume metabolic activity, cell growth, and division. L-alanine is one such potent germinant signal for many bacterial species.^22^^,^^23^^,^^24^ Interestingly, its enantiomer D-alanine inhibits spore germination.^25^^,^^26^ The biological importance of this inhibition is poorly understood.

Here, we examine how L- and D-alanine (L-ala and D-ala) impact *Bacillus subtilis* spores in order to extract a broader conceptual understanding of enantiomer sensing. We mapped the germination response of spores to varying L- and D-ala concentrations and also measured the concentrations produced by different bacterial species. We find that the enantiomer ratios produced by most species hinder *B. subtilis* spore germination. Furthermore, we show that remaining dormant in the presence of other bacterial species provides a survival benefit to spores. Together, our results suggest the alanine enantiomer ratio acts as a social avoidance signal, enabling bacterial spores to avoid germinating in the presence of competing species.

## Results

### Alanine sensing allows for enantiomer-specific spore germination responses

**Figure 1:**
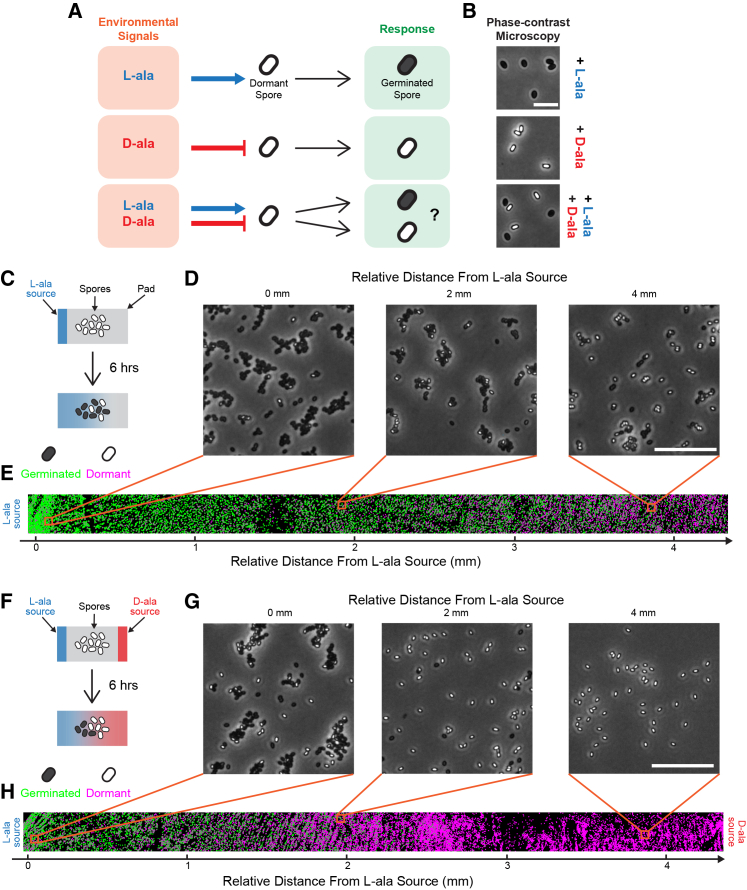
Alanine signaling allows for enantiomer-specific spore germination response (A) Diagram illustrates the response of *B. subtilis* spores to different environmental signals. Spores germinate when exposed to L-ala (top), yet remain dormant when exposed to D-ala (middle). They exhibit a heterogeneous response when exposed to both L- and D-ala (bottom). (B) Phase contrast microscopy images of *B. subtilis* spores 2 h after exposure to 0.1 mM L-ala only (top), 0.2 mM D-ala only (middle), and a mixture of 0.1 mM L- and 0.2 mM D-ala (bottom). Dormant spores are phase-bright, while germinated spores are phase-dark. (C) Schematic of the setup of the spatially varying L-ala experiment. Spores are immobilized underneath an agarose pad. Initially, a strip of agarose containing 0.1 mM L-ala is added on the left side (shown in blue, top). Over 6 h, the L-ala diffuses across the pad, creating a concentration gradient (bottom). (D) Phase-contrast microscopy images of spores at different distances from the L-ala source 6 h after adding the source. (E) Strip image where individual spores are color-coded based on their germination state (dormant-magenta, germinated-green). Locations of images in (D) are indicated with orange boxes. (F) Schematic of the initial setup of the spatially varying L- and D-ala experiment, similar to (C). A strip of agarose containing 0.1 mM L-ala is added on the left side (shown in blue, top), while a strip containing 0.2 mM D-ala is added on the right side (shown in red, top). Over 6 h, both L- and D-ala diffuse into the pad (bottom). (G) Phase contrast microscopy images of spores at different distances from the L-ala source 6 h after adding the L- and D-ala sources. (H) Strip image similar to (E). Scale bars, (B) 5 μm, (D and G) 20 μm.

We first confirmed that spores of a non-domesticated strain of *B. subtilis* (NCIB 3610) use alanine enantiomers as opposing germination signals (Figure 1A). Spores were immobilized on agarose pads, and L-ala and/or D-ala were added to each pad. Spore germination was identified using phase contrast microscopy, where dormant spores are phase-bright, and germinated (hydrated) spores are phase-dark (Figure 1B). As expected, spores germinated when exposed to L-ala (Figures 1A and 1B, top, Video S1), while spores exposed to D-ala remained dormant (Figures 1A and 1B, middle, Video S1). When exposed to both L- and D-ala simultaneously, spores exhibited a heterogeneous germination response, with some spores germinating and others remaining dormant (Figures 1A and 1B, bottom, Video S1). These results are consistent with D-ala inhibiting and L-ala inducing spore germination.

Video S1. Spore germination in response to L- or D-alanine, related to Figure 1 Time-lapse phase contrast microscopy movie of *B. subtilis* spores exposed to top: 0.1 mM L-ala, middle: 0.2 mM D-ala, and bottom: 0.1 mM L-ala +0.2 mM D-ala. Time stamp is in hours: minutes. Scale bars, 5 μm.

We further characterize the opposing roles of alanine enantiomers in terms of sensitivity to signal concentrations. We developed an experiment to spatially vary the L-ala concentration and examine the localized germination response of spores. Specifically, we provided a source of L-alanine on one side of a population of spores by adding a thin strip of agarose containing L-ala (Figure 1C, top). Over time, this L-alanine diffused through the pad, creating a concentration gradient across the spore population (Figure 1C bottom; Figure S1). We then used phase contrast microscopy to monitor spore germination 6 h after the addition of the L-ala source as a function of distance from it. As expected, a higher fraction of spores germinated closer to the L-ala source, while fewer spores germinated farther away from it (Figures 1D, 1E and S1). This data demonstrates that the fraction of spores that germinate is dependent upon the L-ala concentration experienced by the spores, which can give rise to a spatial gradient of germination responses.

Next, we asked if the presence of D-ala could be used to spatially modify the L-ala response. To test the integration of opposing environmental signals, we added a D-ala source to the right-hand side of the pad, keeping the L-ala source on the left as in the previous experiment (Figure 1F, top). The L- and D-ala sources created competing L- and D-ala gradients across the spore population, with higher concentrations of D-ala on the right-hand side of the pad (Figure 1F, bottom). As expected, the D-ala suppressed spore germination on the right-hand side of the spore population (Figures 1G, 1H and S1). This created a steeper response curve in the presence of competing signals when compared to the pad with L-ala only (Figure S1). Taken together, this data shows that spore germination depends on the local concentrations of both L- and D-alanine signals. Alanine enantiomers can thus serve to provide spatial information about the spore environment.

### Bacterial spores integrate the alanine enantiomer ratio to determine when to germinate

**Figure 2:**
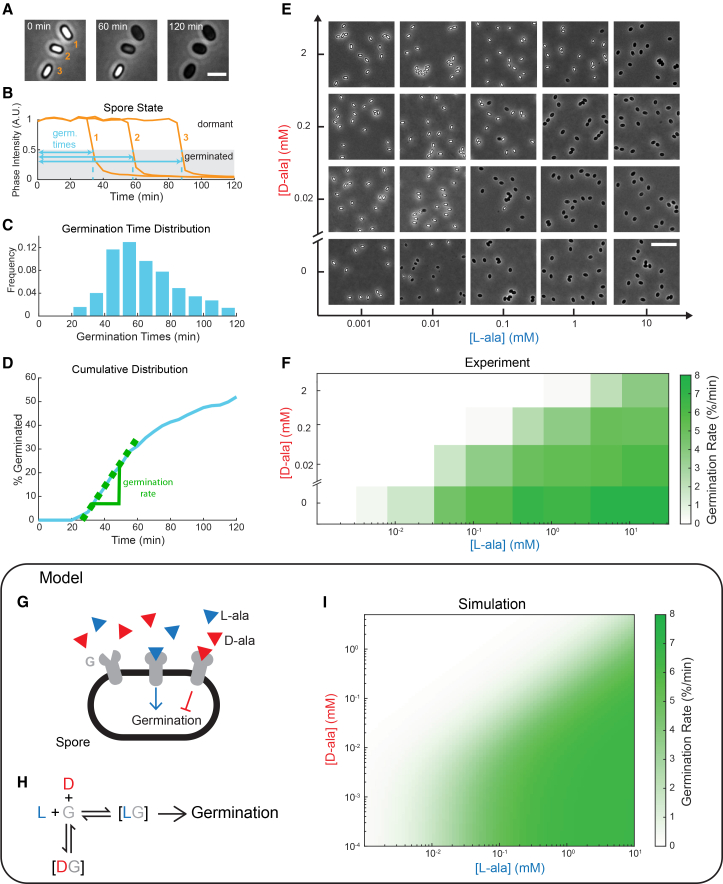
Bacterial spores integrate the alanine enantiomer ratio to determine when to germinate (A) Phase contrast microscopy filmstrip of *B. subtilis* spores. 0.01 mM L-ala is added at 0 min (B) Normalized phase contrast intensity as a function of time for the three spores pictured in (A). The germination time is determined to be when the phase intensity drops below 50% (shown in cyan). (C) Distribution of germination times for *N* > 1000 spores (all exposed to 0.01 mM L-ala). (D) Cumulative distribution of germination times for spores exposed to 0.01 mM L-ala. The maximum germination rate is determined by fitting a line to the largest slope (shown in green). (E) Phase contrast microscopy images of spores 2 h after being exposed to different L- and D-ala concentrations. (F) Enantiomer phase space map of the maximum germination rate as a function of L- and D-ala exposure. (G) Cartoon of molecular components of alanine enantiomer signaling. L-ala (blue triangles) and D-ala (red triangles) both bind to a shared germinant receptor G (shown in gray). L-ala binding causes germination while D-ala binding inhibits germination. (H) Competitive binding scheme of alanine enantiomers (blue L and red D) to the germinant receptor (gray G). (I) Simulated response map created by fitting an equilibrium binding kinetic model for the scheme in panel H, to the measured germination responses in (F), with dissociation constants for L-ala (*k*_*L*_) of 0.026 mM^−1^ and for D-ala (*k*_*d*_) of 0.0052 mM^−1^ (See Methods and Table S1). Scale bars, (A) 2 μm, (E) 10 μm.

We next systematically mapped the germination response of spores to different concentrations of the alanine enantiomers. Using time-lapse phase-contrast microscopy, we imaged the germination state of spores as a function of time (Figure 2A). By tracking the phase contrast of individual spores, we identified their time to germination, as defined by the time between alanine exposure and when the phase intensity drops below 50% (Figure 2B). By tracking hundreds of spores, we determined the distribution of germination times (Figure 2C). We then identified the maximum germination rate from the cumulative distribution of germination times (Figure 2D, Methods). We calculated the germination rate of spores exposed to various L- and D-ala concentration pairs spanning over 4 orders of magnitude (Figures 2E, 2F, S2A and S2B, Video S2). The resulting germination response phase space shows that D-ala reduces spore germination in a concentration-dependent manner, with the response largely determined by the L-to D-ala ratio (Figure S2C).

Video S2. Spore germination in response to different L- and D-alanine concentrations, related to Figure 2 Time-lapse phase contrast microscopy movie of *B. subtilis* spores exposed to noted concentrations of L- and D-ala. Time stamp is in hours: minutes. Scale bars, 10 μm.

To test the sufficiency of the enantiomer integration in governing the germination response, we developed a mathematical model of enantiomer signal integration (Figures 2G–2I). Previous research indicated that L- and D-ala bind competitively to a common germinant receptor, known as GerA^27^ (Figures 2G and 2H). Germination occurs when L-ala binds to the GerA receptor, with D-ala acting as a competitive inhibitor. Using equilibrium binding kinetics, we developed a model that considers the germination rate as proportional to the amount of germinant receptor bound to L-ala (Methods). This model was able to recapitulate the experimentally determined enantiomer response phase space (Figure 2I and Table S1). In the presence of appreciable D-ala ([D-ala]>*k*_*l*_), the L-/D-ala enantiomer ratio is thus sufficient to explain the germination decision by spores.

### Bacteria release alanine enantiomer ratios outside the germination region

**Figure 3:**
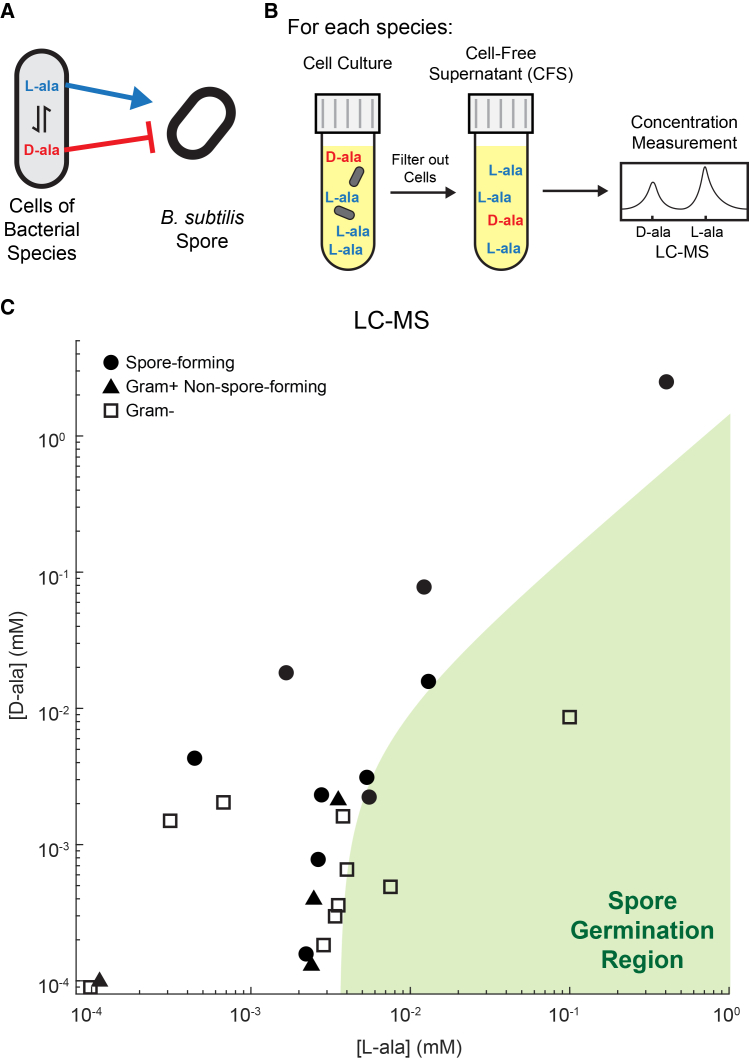
Bacteria largely produce alanine enantiomer ratios that are predicted to maintain dormant spores (A) Cartoon shows that bacteria interconvert L- and D-ala, while also secreting them into their environment, where they can influence nearby spore germination. (B) Schematic of cell-free supernatant (CFS) production from various bacterial species. Following CFS production, the L- and D-ala concentration is measured analytically using LC-MS. (C) L- and D-ala concentrations in CFS from (●) spore-forming, (▲) Gram+ non-spore-forming, and (□) Gram- species. The region causing the germination of *B. subtilis* spores (from the model in Figure 2I) is indicated in green.

While L-ala is used across all the kingdoms of life, D-ala is almost exclusively produced by bacteria, where it is a structural part of the peptidoglycan stem and interpeptide bridge.^3^^,^^4^^,^^28^ D-ala is typically released during peptide crosslinking.^29^ We reasoned that nearby bacterial cells (other than *B. subtilis*) may release alanine into the surrounding environment and play a crucial role in the decision of *B. subtilis* spores to germinate. We thus asked how the L- and D-ala produced and secreted by other bacterial species affects *B. subtilis* spores (Figure 3A).

To address this question, we identified 24 diverse bacterial species capable of aerobically growing in a minimal medium (Table S2) that contains no L- or D-ala, and does not elicit *B. subtilis* spore germination (Figure S3). Given that many bacterial species release D-amino acids (including D-ala) during the stationary phase,^19^ we grew each of these species to the stationary phase. We then removed cells by centrifugation followed by filtration, isolating secreted factors in the cell-free supernatant (CFS). We analytically measured the L- and D-ala concentrations in CFS from each of the species by reaction with a chiral derivatizing agent followed by liquid chromatography-tandem mass spectrometry (LC-MS) (Figure 3B and Table S3, see Methods for details). Surprisingly, while L- and D-ala concentrations in the CFS spanned over 4 orders of magnitude, most of the species created alanine enantiomer ratios that fall just outside the previously mapped germination response region (Figures 3C, S4A, S4B and S5). *B. subtilis* spores should thus not germinate in the presence of most of these other bacterial species.

### Spores remain dormant when exposed to cell-free supernatant from other bacterial species

**Figure 4:**
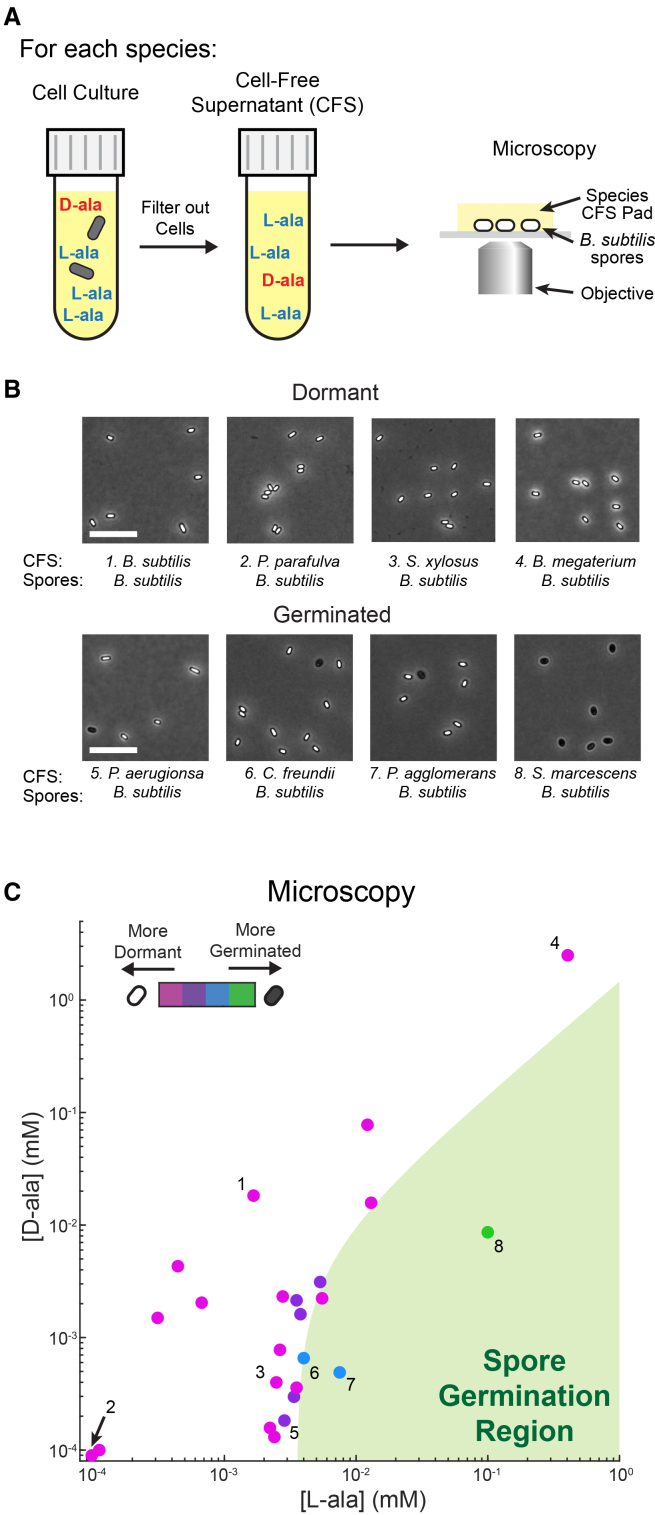
Spores remain dormant when exposed to CFS from other bacterial species (A) Schematic of cell-free supernatant (CFS) production from various bacterial species. Following CFS production, the germination response of *B. subtilis* spores is measured using phase contrast microscopy. (B) Representative phase contrast images of *B. subtilis* spores on CFS from bacterial species that maintain dormancy (top) and induce germination (bottom). See Figures S4A and S4B for images of all species tested. (C) Relative germination of *B. subtilis* spores on CFS from each species after 2 h is indicated using a magenta-to-green color scale (as measured using phase-contrast microscopy). The species shown in (B) are indicated using their corresponding number. As in Figure 3C, each species dot is placed using L- and D-ala concentration measurements from LC-MS, and the region causing the germination of *B. subtilis* spores (from the model in Figure 2I) is indicated in green. Scale bars, 10 μm.

Next, we directly tested how *B. subtilis* spore germination was affected by CFS from other species. *S*pores were placed on agarose pads that contained CFS from one of the different species, and spore germination was monitored by phase-contrast microscopy (Figure 4A). By using CFS pads, we were able to image individual spore responses as before. Consistent with the analytical measurements described above, *B. subtilis* spores remained dormant on CFS from most species (Figure 4B, top), with spores exhibiting significant germination on CFS from only a few species (Figure 4B, bottom; Figures S4A and S4B). Specifically, only ∼15% of the species tested (3 of 24) elicited meaningful spore germination (affecting more than 10% of *B. subtilis* spores) after 2 h, while ∼65% of the species (16 of 24) elicited only minimal germination (limited to less than 2.5% of spores) (Figures S4A–S4C).

We find agreement between the degree of *B. subtilis* spore germination measured by microscopy on pads with CFS from each species (magenta-to-green color scale, Figures 4C and S4C) and the analytically measured L- and D-ala concentrations in the CFS for each species (Figure S5). The deletion of the germinant receptor *gerA* abolished spore germination in CFS from the few outlier species that induced moderate *B. subtilis* spore germination (Figures S6A and S6B), suggesting L-Ala as the germination trigger (See limitations of the study). Conversely, we confirmed that the inhibition of spore germination in CFS was due to D-ala. We used D-amino acid oxidase to enzymatically degrade D-ala in CFS, which as expected resulted in *B. subtilis* spore germination (Figures S7A and S7B). Together, these data demonstrate that most bacterial species secrete alanine enantiomer ratios under which *B. subtilis* spores remain dormant. *B. subtilis* spores thus appear to integrate their environmental alanine enantiomer ratio to possibly sense other species and govern their germination decision.

### Social avoidance provides a competitive advantage to B. subtilis spores

**Figure 5:**
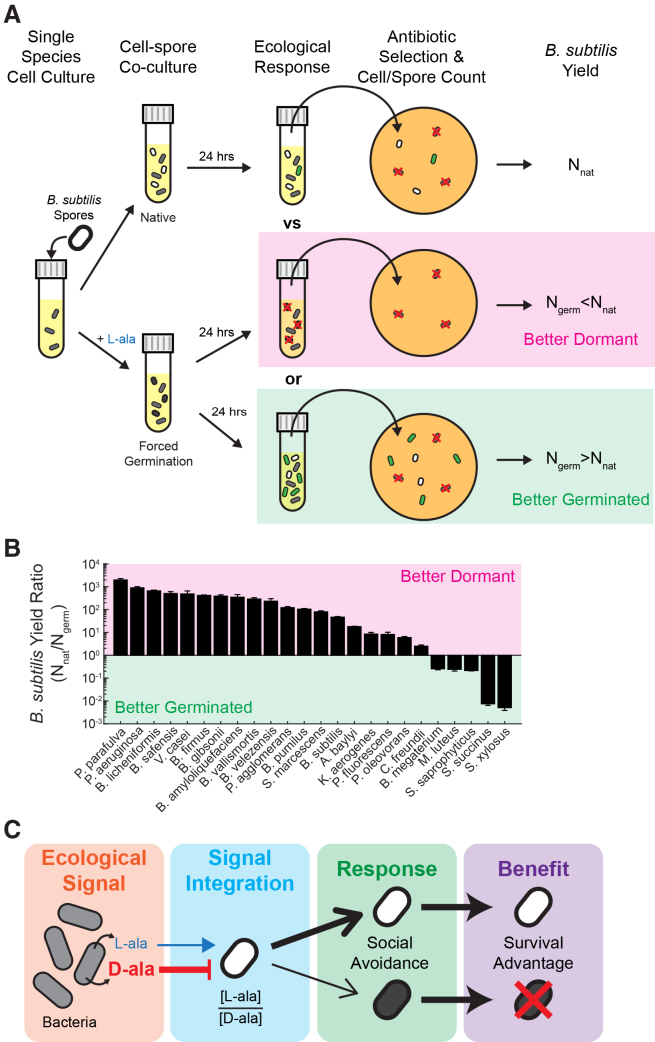
Social avoidance provides a competitive advantage to *B. subtilis* spores (A) Cartoon of the competition assay. *B. subtilis* spores (white) are added to a stationary phase culture of each species (gray). In the native arm, the cell-spore co-culture incubates without the addition of exogenous L-ala (top). In the perturbation arm, 0.5 mM L-ala is added to the cell-spore co-culture to force the germination of the *B. subtilis* spores (black), followed by a 24-h incubation. For each condition, the number of viable *B. subtilis* cells/spores is determined using colony-forming units (CFUs) on plates with antibiotic selection specific for the added *B. subtilis* spores. The number of viable *B. subtilis* under native conditions (N_nat_, top row) serves as a comparison. If the spores are better remaining dormant, they may experience cell death (red “x’s”) when forced to germinate, leading to less viable *B. subtilis* (N_germ_<N_nat_, middle). Alternatively, if the spores are better germinating, they will outgrow and proliferate (green), leading to more viable *B. subtilis* (N_germ_>N_nat_, bottom). (B) *B. subtilis* yield ratio, defined as the ratio of N_nat_/N_germ_, for all species tested. Values greater than 1 indicate a competitive advantage for the spores remaining dormant, while values less than 1 indicate a competitive advantage for spores germinating. Data are represented as mean ± SEM. (C) Summary of alanine enantiomer signaling in *B. subtilis* spores. Nearby bacterial species release L- and D-ala. Spores integrate the enantiomers, detecting their ratio. Most species release alanine enantiomer ratios that cause spores to remain largely dormant. This social avoidance imparts a benefit for *B. subtilis*, as the spores are unable to outgrow and die in the presence of most other species.

Our findings raise the question as to why *B. subtilis* spores predominantly remain dormant in the presence of other bacterial species? We reasoned that enantiomer ratio integration could serve as a social avoidance mechanism that provides a competitive advantage for spores. In other words, we hypothesize that *B. subtilis* spores use alanine enantiomer ratios to probe the ecological makeup of their environment, in order to decide whether it is advantageous for them to germinate. This conjecture assumes that the presence of other bacterial species would be detrimental to the survival of recently germinated *B. subtilis* cells. To test this hypothesis, we developed a direct competition assay to test how well germinated *B. subtilis* spores survive in the presence of cells from another bacterial species. Specifically, we forced *B. subtilis* spores to germinate using *exogenous* L-ala addition (0.5 mM) and performed pairwise competition assays between *B. subtilis* spores and cell cultures of each tested species. After 24 h of incubation, the *B. subtilis* yield was determined in each of the co-cultures (Figure 5A, see Methods for details). The yield without *exogenous* L-ala addition (N_nat_) provided a baseline for how *B. subtilis* spores fared under unforced (native) germination conditions.

If cells arising from forced germination are unable to grow (or even die) when competing with another species, the *B. subtilis* yield would be lower compared to the natural (unforced) condition (N_germ_<N_nat_, Figure 5A, middle pink-shadowed row). Alternatively, if spores are better off germinating in the presence of another competing species, they should be able to grow and divide, resulting in a higher yield compared to the native (unforced) condition (N_germ_>N_nat_, Figure 5A, bottom green-shadowed row). We found that in the majority (∼80%) of co-cultures, forced germination resulted in less viable *B. subtilis* (Figure 5B), indicating that the spores are better off remaining dormant. In the cases where forced germination provided an advantage in co-cultures, there was no clear association with the L- and D-alanine levels secreted by the other species, nor did these species show significant germination of *B. subtilis* spores (Figure S8). Collectively, these results suggest that dormancy confers a competitive advantage to *B. subtilis* spores in most inter-species microbial interactions. *B. subtilis* spores are thus better off not germinating in the majority of co-cultures, and those spores that did germinate in the presence of another species were not only unable to compete, but also more likely to die (Figure 5C).

## Discussion

In this study, we demonstrate that different bacterial species produce unique concentrations of L- and D-ala, and that *B. subtilis* spores are able to ratiometrically detect these signals. Such ratiometric responses have been identified in other biological systems,^30^^,^^31^^,^^32^^,^^33^ suggesting they are a common method for integrating positive and negative signals. For the spore, ratiometric sensing via competitive binding to a common receptor allows the spore to integrate signals using limited resources. Because this signal integration mechanism does not require regulation via transcription or signaling cascades, it does not require metabolic activity or energy, both of which are strongly limited in spores.

Spore germination inhibition by D-ala produced by nearby bacterial species is an important signal leading spores to remain dormant. Our data shows that this maintenance of dormant spores in the presence of nearby bacterial species provides a direct survival benefit to the spore. This is likely because the outgrowth of spores is costly, and they may not be able to compete with an already active cell. This survival benefit suggests an advantage to the *spore* for alanine-mediated social avoidance. Of course, maintenance of dormant spores may also provide a benefit to nearby vegetative *cells* of other bacterial species, as they do not have to compete with *B. subtilis* spores. This benefit could be due to limited competition for resources and space, or a reduction in natural antibiotic production. Thus, alanine-mediated social avoidance extends the view that spore formation is a community behavior that influences natural ecosystems,^34^^,^^35^ and future work should examine the impacts of this social avoidance on bacterial ecosystems.

Finally, our work sheds light on the importance of the molecular handedness of amino acids in ecosystems. While life on planet Earth predominantly is based on L-amino acids, some microbes can produce D-amino acids. Many studies have investigated the importance of enantiomers in biological systems, in particular the role of D-amino acids.^15^^,^^16^^,^^19^^,^^36^ While historically D-alanine is recognized for its structural role in the bacterial cell wall, recent studies demonstrate D-alanine plays a role in regulating a variety of processes, including host-microbe interactions^7^^,^^37^ and plant growth.^38^^,^^39^ As microbes are the predominant source of D-amino acids, they provide a bacteria-specific signal. Our work provides additional insights by revealing that bacteria use amino acids handedness as a means to sense nearby bacterial species and possibly exert control over each other. It is thus intriguing to consider the importance of handedness in the ecological context, as it appears to serve as a unique inter-species and inter-kingdom signal.

### Limitations of the study

In this study, we focus on alanine enantiomer signaling; however, multiple other germination pathways exist in *B. subtilis* spores,^40^^,^^41^ as well as other spore species.^42^ The CFS used in this study likely includes a complex mixture of compounds, including other amino acids, nucleosides, and sugars that can act as germinants.^43^^,^^44^^,^^45^ Low levels of spore germination on CFS from some species may be due to the presence of some of these alternative germinants. Similarly, reduced germination in yet other species CFS may indicate the presence of germination inhibitors such as alcohols or fatty acids.^44^^,^^46^ Despite these limitations, our results show that *B. subtilis* spores avoid germination in the presence of most species and that this social avoidance provides a survival advantage.

## Resource availability

### Lead contact

Requests for resources and reagents should be directed to Gürol Süel (gsuel@ucsd.edu).

### Materials availability

This study did not generate new unique reagents.

### Data and code availability

• All data are reported within the article and supplemental information and will be shared by the lead contact upon request.
• All original code has been deposited at Zenodo and is publicly available at Zenodo: https://doi.org/10.5281/zenodo.18341415 as of the date of publication.
• Any additional information required to reanalyze the data reported in this article is available from the lead contact upon request.

## Acknowledgments

We thank Jeff Hasty, Kit Pogliano, and Rachel Dutton for bacterial strains used in this work. We thank Munehiro Asally, Leticia Galera-Laporta, and members of the Suel Lab for useful discussions.

This work was supported by the 10.13039/100000057National Institute of General Medical Sciences [grant R35 GM139645 (G.M.S.)], the Army Research Office [grants W911NF-22-1-0107 and W911NF-1-0361 (G.M.S.)], the 10.13039/100000865Bill & Melinda Gates Foundation INV-067331 (G.M.S.), the Spanish Ministry of Science, Innovation and Universities and 10.13039/501100002924FEDER projects PID2021-127311NB-I00 and CEX2018-000792-M (J.G.-O.), and the Generalitat de Catalunya ICREA Academia program (J.G.-O.).

## Author contributions

Conceptualization, C.J.C., T. K.-T.C., J.G.-O., and G.M.S.; data curation, C.J.C.; formal analysis, C.J.C.; funding acquisition, J.G.-O. and G.M.S.; investigation, C.J.C., T.K.-T.C., R.A., and M.B.; methodology, C.J.C., T.K.-T.C., R.A., J.G.-O., and G.M.S.; project administration, G.M.S.; supervision, G.M.S.; visualization, C.J.C., T. K.-T.C., J.G.-O., and G.M.S.; writing – original draft, C.J.C. and G.M.S.; writing – review and editing, C.J.C., T.K.-T.C., R.A., M.B., J.G.-O., and G.M.S.

## Declaration of interests

The authors declare no competing interests.

## STAR★Methods

### Key resources table

**Table:** 

REAGENT or RESOURCE	SOURCE	IDENTIFIER
**Bacterial strains**		

*B. subtilis* NCIB 3610	Wade Winkler laboratory^47^	NCIB 3610
*B. subtilis* NCIB 3610 *amyE: Phyp-YFP*	This paper	–
*Bacillus licheniformis*	Kit Pogliano laboratory	–
*Bacillus amyloliquefaciens*	Kit Pogliano laboratory	–
*Bacillus gibsonii*	Provided by AGBiome	–
*Bacillus vallismortis*	Provided by AGBiome	–
*Bacillus velezensis*	Provided by AGBiome	–
*Bacillus firmus*	Kit Pogliano laboratory	–
*Bacillus megaterium*	Kit Pogliano laboratory	–
*Bacillus safensis*	Provided by AGBiome	–
*Bacillus pumilus*	Kit Pogliano laboratory	–
*Micrococcus luteus*	Kit Pogliano laboratory	–
*Staphylococcus xylosus*	Rachel Dutton laboratoy	–
*Staphylococcus saprophyticus*	Rachel Dutton laboratoy	–
*Staphylococcus succinus*	Rachel Dutton laboratoy	–
*Pseudomonas aeruginosa*	Suel Lab library	–
*Klebsiella aerogenes*	Suel Lab library	–
*Pseudomonas fluorescens*	Suel Lab library	–
*Citrobacter freundii*	Suel Lab library	–
*Serratia marcescens*	Suel Lab library	–
*Pantoea agglomerans*	Kit Pogliano laboratory	–
*Vibrio casei*	Rachel Dutton laboratoy	–
*Pseudomonas parafulva*	Kit Pogliano laboratory	–
*Pseudomonas oleovorans*	Jeff Hasty laboratory	–
*Acinetobacter baylyi*	Jeff Hasty laboratory	–

**Chemicals, peptides, and recombinant proteins**		

Glycerol	Sigma-Aldrich	Cat#G5516, CAS: 56-81-5
L-glutamic acid monosodium salt hydrate (anhydrous)	Sigma-Aldrich	Cat#G5889, CAS: 142-47-2
Magnesium chloride hexahydrate	Fisher Scientific	Cat#BP214, CAS: 7786-30-3
Potassium phosphate monobasic	Fisher Scientific	Cat#BP362, CAS: 7778-77-0
Potassium phosphate dibasic	Fisher Scientific	Cat#BP363, CAS: 7758-11-4
Thiamine HCl	Fisher Scientific	Cat#BP892, CAS: 67-03-8
Manganese chloride	Acros Organics	Cat#AC193451000, CAS: 13446-34-9
Calcium chloride	Fisher Scientific	Cat#BP510, CAS: 10035-04-8
Iron (III) chloride	Acros Organics	Cat#AC217090025, CAS: 10025-77-1
Zinc (II) chloride	Sigma-Aldrich	Cat#Z0152, CAS: 7646-85-7
MOPS	Sigma-Aldrich	Cat#M3183, CAS: 1132-61-2
Potassium chloride	Sigma-Aldrich	Cat#P3911, CAS: 7447-40-7
Sodium chloride	Fisher Scientific	Cat#BP358-1, CAS: 7647-14-5
Ammonium chloride	Fisher Scientific	Cat#A661, CAS: 12125-02-9
Sodium sulfate	Fisher Scientific	Cat#BP354, CAS: 7757-82-6
Ammonium nitrate	Fisher Scientific	Cat#A676, CAS: 6484-52-2
Magnesium sulfate	Fisher Scientific	Cat#M65, CAS: 7487-88-9
Spectinomycin	Sigma-Aldrich	Cat#S4014, CAS: 22189-32-8
D-amino acid oxidase from porcine kidney	Sigma-Aldrich	Cat#A5222, CAS: 9000-88-8
Flavin adenine dinucleotide	Sigma-Aldrich	Cat#F8384, CAS: 84366-81-4
L-alanine	Thermo Scientific	Cat#J60279.22, CAS: 56-41-7
D-alanine	Sigma-Aldrich	Cat#A7377, CAS: 338-69-2
Agarose, Low Melting Point	Promega	Cat#V2111, CAS: 9012-36-6

**Software and algorithms**		

MATLAB	MathWorks, 2023	https://www.mathworks.com/products/matlab.html
FIJI	Schindelin et al., 2012^48^	https://fiji.sc/
Original Code	This paper	Zenodo: https://doi.org/10.5281/zenodo.18341415
MetaMorph Microscopy Automation and Image Analysis Software	Molecular Devices, LLC	https://www.moleculardevices.com/products/cellular-imaging-systems/high-content-analysis/metamorph-microscopy
Olympus FluoView (FV31S)	Evident	https://evidentscientific.com/en/products/obsolete/fv3000

**Other**		

Glass-bottom dish	Ted Pella	Cat#14027-20

### Experimental model and study participant details

#### Bacterial strains

Wild-type *Bacillus subtilis* NCIB 3610 was a gift from W. Winkler.^47^ For the co-culture competition assay, *Bacillus subtilis* NCIB 3610 *amyE::P*_*hyp*_*-YFP*(Spec^R^) was used. For integration at the *amyE* locus, the pDL30 plasmid (kind gift from Jonathan Dworkin, Columbia University) was used. Transformation was conducted using a standard one-step transformation procedure.^49^ Chromosomal integration was confirmed using colony PCR. Other bacterial species used are detailed in Table S2. Species were confirmed using 16S rRNA gene sequencing.

#### Growth conditions and media

Prior to all experiments, bacteria were streaked onto Luria Broth (LB) agar plates from glycerol stocks stored at -80°C. LB plates contained antibiotics for the selection of *B. subtilis* transformants when necessary (300 μg ml^-1^ spectinomycin). Plates were incubated overnight at the appropriate temperature for each species (Table S2). Single colonies were always inoculated in liquid LB and incubated at the appropriate temperature in an orbital shaking incubator (200 rpm) for at least 2 hrs before transferring to other media.

Resuspension medium (RM) was used for the preparation of *B. subtilis* spores and mapping of spore response to alanine enantiomers. 1 L of RM contains 46 μg FeCl_3_, 4.8 g MgSO_4_, 12.6 mg MnCl_2_, 535 mg NH_4_Cl, 106 mg Na_2_SO_4_, 68 mg KH_2_PO_4_, 96.5 mg NH_4_NO_3_, 219 mg CaCl_2_, and 2 g monosodium L-glutamate.^50^

All cell-free supernatants were prepared in MSgg containing 5 mM potassium phosphate buffer (pH 7.0), 100 mM MOPS buffer (pH 7.0, adjusted using NaOH), 2 mM MgCl_2_, 700 μM CaCl_2_, 50 μM MnCl_2_, 100 μM FeCl_3_, 1 μM ZnCl_2_, 2 μM thiamine HCl, 0.5% (v/v) glycerol and 0.5% (w/v) monosodium L-glutamate.

### Method details

#### Spore preparation

Spores were prepared using the Resuspension Method.^50^ A 5 mL liquid LB culture was inoculated using a single *B. subtilis* colony, and incubated in an orbital shaker overnight (37°C, 200 rpm). The culture was diluted at an OD_600nm_ 0.1 in 20% (v/v) LB and incubated at 37°C with shaking until reaching an OD_600nm_ 0.6-0.8. The culture was centrifuged at 4,000 rpm for 10 min, and the supernatant was discarded. The cells were resuspended in 1 volume of pre-warmed RM and incubated at 37°C in an orbital shaking incubator (200 rpm) for three days. The resulting spores were spun down at 4,000 rpm for 10 minutes and washed three times with distilled water. Spore suspensions in distilled water were stored at 4°C prior to experiments.

#### Species selection

Species were identified from a lab library containing >50 bacterial species (predominantly found in soil/plant ecosystems and likely to be found in competition with *B. subtilis*). To ensure sufficient growth of each species during CFS preparation, growth was first analyzed on MSgg agar plates incubated up to 3 days at 30 or 37°C, depending on suggested culture conditions for the species. Species that exhibited visible colonies on MSgg agar plates were then grown in liquid MSgg. Cells from a liquid LB starter culture were spun down at 4800 rpm for 2.5 minutes, washed one time in MSgg, and resuspended in MSgg at an OD_600nm_ 0.1. Each species was incubated at the appropriate temperature in an orbital shaker (200 rpm) for 24 hours before measuring OD_600nm_. Only species with OD_600nm_>1.0 were used for CFS preparation.

#### Cell-free supernatant (CFS) preparation

Cells from a liquid LB culture were spun down at 4000 rpm for 4 minutes, washed one time in MSgg, and resuspended in MSgg at an OD_600nm_ 0.1. Each species was incubated at the appropriate temperature (Table S2) in an orbital shaker (200 rpm) for 24 hours. For most species, cultures were spun down at 4000 rpm for 10 minutes, and then the supernatant was filtered using a 0.22 μm filter. For *B. pumilus, B. safensis,* and *B. licheniformis,* the cultures were spun down similarly, and then the supernatant was removed and spun down a second time. The resulting supernatant was then filtered with a 5 μm filter prior to using a 0.22 μm filter. For *B. firmus* and *S. marcescens*, the cultures were spun down at 4000 rpm for 10 minutes, and the resulting supernatant was further spun down for 15 minutes at 5000 rpm. The resulting supernatant was then filtered with a 5 μm filter prior to using a 0.22 μm filter. CFS was immediately aliquoted and stored at -80°C.

For treatment of CFS with D-amino acid oxidase (DAAO), 50 uM flavin adenine dinucleotide (FAD) and 1 U/mL of DAAO was added to freshly made CFS. A control sample was made by adding FAD to the same batch of CFS. The samples were incubated at 37°C in a cell culture tube with shaking for 120 minutes before immediately making agarose pads as described below.

#### Microscopy sample preparation

Agarose pads were made by mixing 1.5% (w/v) low-melting point agarose with either RM (Figures 1 and 2), or the specified CFS (Figure 3). Where necessary, D-alanine was added. The agarose was melted using a standard microwave, bring the mixture just to a boil two times with mixing in between. Pads were poured by pipetting 16 mL of the hot agarose mixture into a petri dish. The agarose was allowed to cool for 15-30 minutes at room temperature, before allowing the plate to further solidify in a 30°C incubator overnight. The next day, pads were cut at the desired size, and a small amount of spore suspension was added to the top of the pad. Once the spore suspension had dried, the pad was inverted into a WillCo glass bottomed dish.

For spatial environment imaging (Figures 1C–1L), an agarose pad (without spores) with 0.1 mM L-ala or 0.2 mM D-ala was added to the top of each agarose pad on the left- and right-hand side prior to starting microscopy imaging. For spore germination response imaging (Figures 1B, 2A and 2E) a 100× solution of L-ala was added to the top of each pad.

#### Microscopy

Phase-contrast microscopy images of spore germination were obtained using a widefield Olympus IX83 inverted epifluorescence microscope or an Olympus FV3000 inverted confocal microscope. Widefield images were acquired using either a UPLFLN40XPH objective (for strip images in Figures 1C–1H), or a UPLXAPO100XOPH objective (Figures 1B, 2 and 3), an Orca-Fusion BT camera (Hamamatsu), and MetaMorph Imaging Software (Molecular Devices). Focus was maintained using software autofocus. 40X strip images were acquired with ∼10% overlap between adjacent images. Strip images were acquired at 15-minute intervals. 100X images were acquired at 5-minute intervals. Confocal images were acquired using a UPLanFLN60X objective and FluoView imaging software with drift compensation. Images were acquired with a scanning speed of 4 μs/pixel and a pixel size of 103.6 nm. Images were acquired at 5-minute intervals. All microscopy was conducted inside an incubated chamber maintained at 30°C.

### Quantification and statistical analysis

#### Spatial alanine germination image analysis

Strip images were created and processed using custom MATLAB software. Sequential images were acquired with ∼10% overlap. SIFT features were used for both image alignment (in time) and stitching (in space). First, the initial time point and 6-hour timepoint for each imaging location were aligned. A distance filter was used to limit matching SIFT features to a local neighborhood. Next, the sequential images were stitched together. SIFT features were limited to the corresponding edges (∼10% overlap) of the images to ensure proper stitching.

Next, binary maps for dormant and germinated spores were created. Spores were identified from the initial time point image. To do this, the image was sharpened using the unsharp masking technique. Spores were identified using an intensity threshold, and filtered based on size and mean intensity. Next, spore germination was determined by examining the difference in phase intensity between the spore in the initial time point and at 6 hours. To facilitate visualization of the final color-coded strip image, the binary spore maps were dilated using a disk of radius 20 pixels. After dilation, some spore maps overlapped with nearby spores. If these spores had a different germination status, the individual pixels in the overlapping region were randomly assigned to either germinated or dormant. Finally, the dormant and germinated spore maps were combined using a magenta-green colormap.

The fraction of germinated spores as a function of distance (Figure S1) was determined using randomly selected regions at 1 mm intervals. Spore germination was determined as described in “Germination rate analysis” below.

#### Spatial alanine gradient simulation

The gradient of L- and D-alanine concentrations was simulated using the finite difference method to solve a 1D diffusion equation. A diffusion coefficient of 1 × 10^-5^ cm^2^ s^-1^ is assumed to be the same for both L- and D-ala.^51^ The 2 cm agarose pad was discretized in 0.005 cm bins, and a time step of 1 second was used. The L- and D-ala sources were added as additional pad length to the left and right sides. Microscopy images were used to calibrate the distance from the edge of the pad to the imaged region in the experiment, and these values were used to plot the approximate gradient in the imaged region.

#### Germination rate analysis

The phase-contrast intensity of individual spores was tracked using Fiji (https://imagej.nih.gov/ij/, RRID: SCR_002285), similar to described previously.^52^ Small drift in images was corrected using the MultiStackReg plugin (http://bradbusse.net/sciencedownloads.html, RRID: SCR_016098). Single spores were segmented from the first timepoint based on an intensity threshold (Otsu method). Possible spores were filtered using size, shape, and manual inspection. The phase-contrast intensity of individual spores through time was measured using the MultiMeasure plugin (https://www.optinav.info/Multi-Measure.htm).

Spore germination times and maximum germination rates were determined using custom MATLAB software. First, germinated spores were identified using an intensity threshold. The germination time for each spore was then identified as the timepoint when the phase intensity dropped below the midpoint between the intensity prior to germination and after germinating (Figure 2B). The max germination rate was determined by fitting a line to 3 timepoints in the CDF (Figures 2D and S2A).

#### Model

We consider that L-alanine (L) and D-alanine (D) compete for binding to the germinant receptor GerA (G), but only L-alanine triggers a signaling output (S) when bound to the receptor:

L+G⇌[LG]↪S

D+G⇌[DG]

Note that signaling is activated upon binding of L-ala to the receptor, but does not change chemical concentrations. Since the signaling output is much slower than receptor binding,^53^ the reactions operate in equilibrium,

$$L\cdot G=k_{l}\left[\;\mathrm{LG}\right]$$

$$D\cdot G=k_{d}\left[DG\right]$$

where *k*_*l*_ and *k*_*d*_ are the dissociation constants corresponding to the two binding reactions. Considering now that the germinant obeys the conservation law *G*+[*LG*]+[*DG*] = *G*_*T*_ = *constant*, we can solve the equations above to find the steady state concentration of the [*LG*] complex:

$$\left[\mathrm{LG}\right]=\frac{G_{T}L/k_{l}}{1+L/k_{l}+D/k_{d}}$$

Finally, taking into account that the activity of the receptor is proportional to the concentration of the [*LG*] complex, we get to:

$$\frac{dS}{dt}=\frac{\alpha \cdot L}{k_{l}+L+g\cdot D}$$

where *g* = *k*_*l*_/*k*_*d*_ is the ratio between the dissociation constants of L-alanine and D-alanine to the receptor (the higher *g*, the tighter the association between D-alanine and the receptor in comparison with that of L-alanine, for instance). We consider that the germination rate is proportional to the signaling rate *dS*/*dt* above.

The parameter values were determined via least squares fitting to the experimental data. Parameter values are in reasonable agreement with previous measurements^26^ (Table S1).

#### LC-MS

Analytical measurement of L- and D-ala concentrations in CFS were performed by AxisPharm, LLC using methods adapted from previous studies.^54^ Briefly, samples were derivatized by adding 10 μL of 6% triethylamine and 10 μL of a solution of (S)-N-(4-nitrophenoxycarbonyl)phenylalanine methoxyethyl ester (S-NIFE) to a 20 μL aliquot of CFS, and the mixture was allowed to react at room temperature for 20 minutes. The reaction mixture was diluted with 10 μL of 5% acetic acid and an aliquot (20 μL) was analyzed by liquid chromatography tandem mass spectrometry (LC-MS/MS).

#### Cell-spore co-culture competition assay

Liquid LB starter cultures of each species were spun down at 4800 rpm for 2.5 minutes, and washed once in MSgg. They were then resuspended in MSgg at an OD_600nm_ of 0.1. 180 μL of cell culture was added to each well of a 96 well plate (4 wells per species) and the plate was incubated in a Tecan Infinite M Plex plate reader at 30°C with shaking for 24 hours. P_hyp_-YFP *B. subtilis* spores were spun down at 4000 rpm for 10 minutes and resuspended in MS (MSgg lacking glutamate and glycerol) with 10 mM IPTG. The spore suspension was added to each cell culture well (20 μL spores to 180 μL of cell culture). For each species, 0.5 mM of L-ala was added to 2 of the wells to force spore germination. The cell-spore co-cultures were then incubated for a further 24 hours in the plate reader at 30°C with shaking. After 24 hours, the cell-spore co-cultures were serially diluted in MS and plated on LB agar plates supplemented with spectinomycin. The antibiotic selection ensures growth of only the *B. subtilis* spores, while suppressing growth of the competing species. The rich LB media induces germination of any *B. subtilis* spores. Thus, colonies indicate the presence of either a viable *B. subtilis* cell resulting from outgrowth from an added spore or a *B. subtilis* spore. Plates were incubated at 37°C overnight, and colonies were counted by hand.

#### Statistical analyses

No statistical analyses were used in this study. All error bars and sample sizes are defined in the figure legends.
