# Supplementary material for: Enantiomer sensing enables social avoidance by bacterial spores
Source: iScience. 2026 Feb 10;29(3):114967. doi: 10.1016/j.isci.2026.114967 (PMC13080479; doi:10.1016/j.isci.2026.114967)
Supplement: Document S1. Figures S1–S8, Tables S1–S3 [file mmc1.pdf]

iScience, Volume *29*

Supplemental information

Enantiomer sensing enables social avoidance by bacterial spores

Colin J. Comerci, Todd Kwang-Tao Chou, Ramina Amino, Maja Bialecka, Jordi Garcia-Ojalvo, and Gürol M. Süel

Figure S1

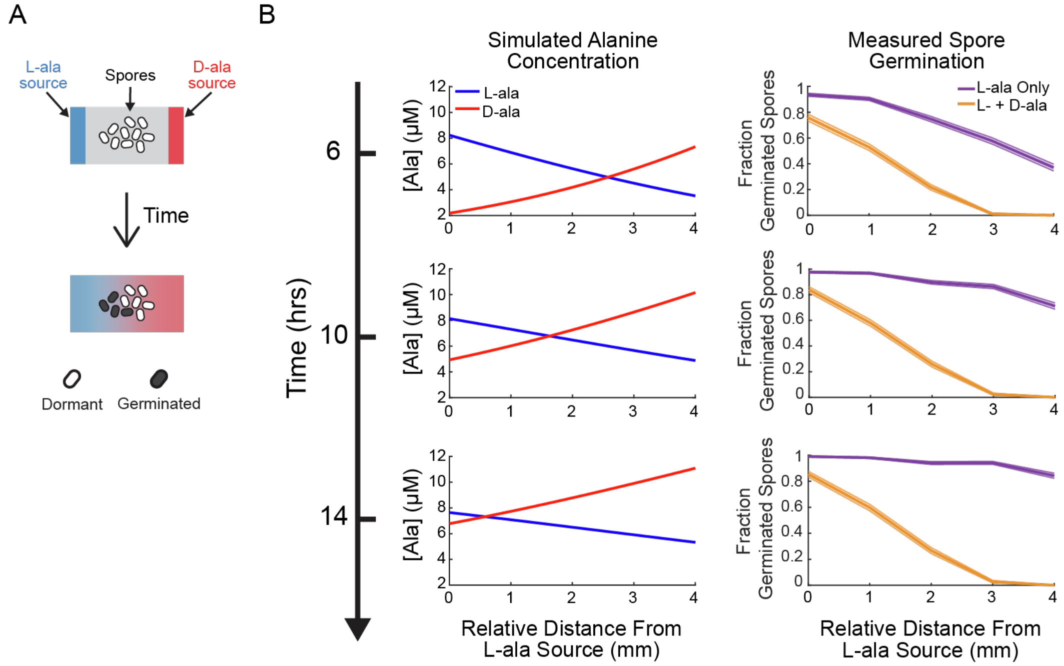

Figure S1. Spatially varying spore germination response, related to Figure 1. (**A**) Schematic of initial setup of spatially varying L- and D-ala experiment. A strip of agarose containing 0.1 mM L-ala is added on the left side (shown in blue, top), while a strip containing 0.2 mM D-ala is added on the right side (shown in red, top). After 6 hours, both L- and D-ala diffuse into the pad (bottom). (**B**) Alanine enantiomer concentration gradients (left column) and spore germination responses (right column) as a function of time since addition of L- and D-ala sources. Left: L- and D-ala concentration gradient (in blue and red, respectively) across the imaged region. Concentrations are determined using a 1D diffusion model (see methods). Right: Measured spore germination fraction when only an L-ala source (purple) and both an L- and D-ala source are added (orange). Error bars represent the mean ± SEM of N>250 spores at each position.

Figure S2

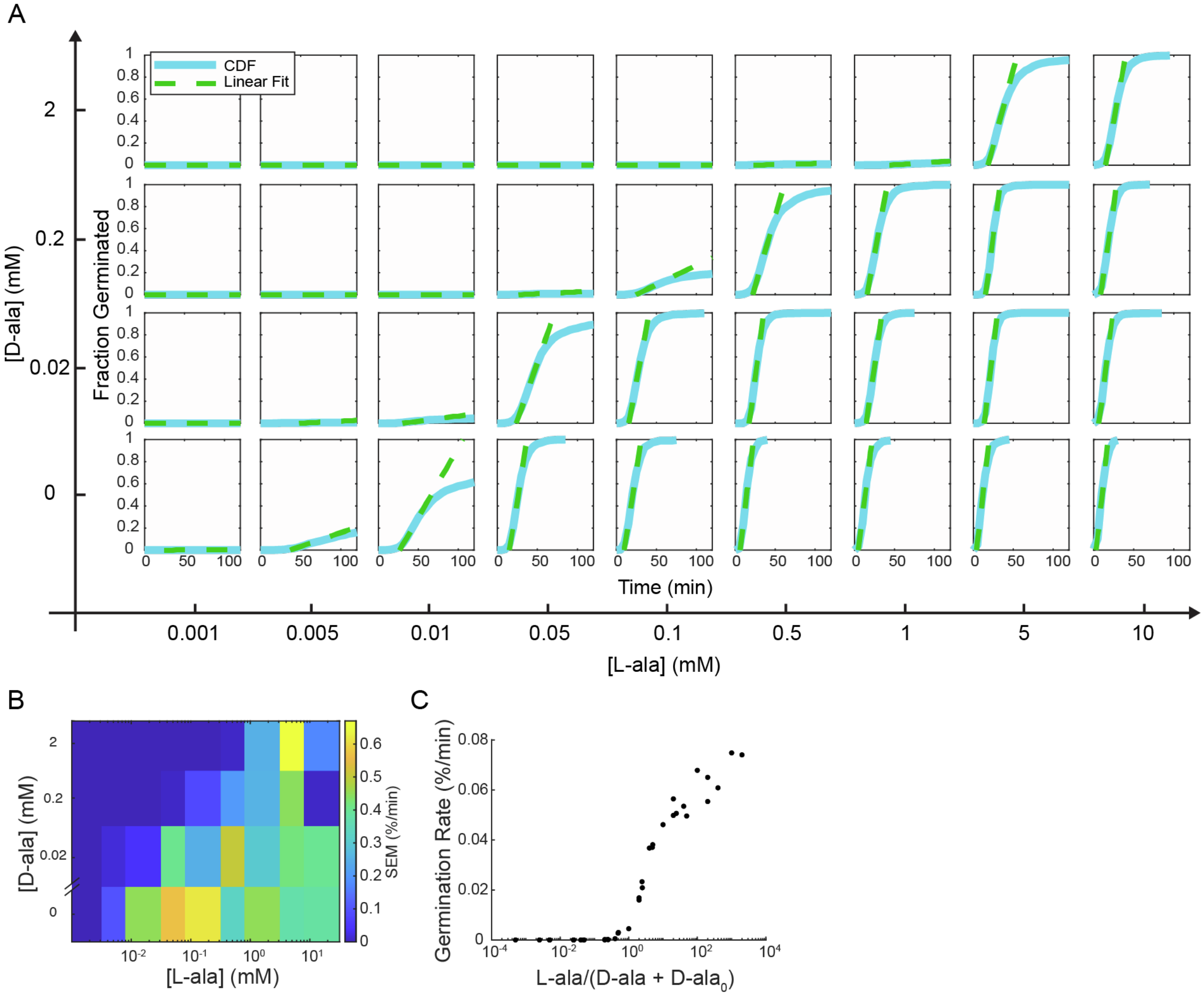

Figure S2. Germination response phase space for alanine enantiomers, related to Figure 2. (**A**) Cumulative distribution function (CDF, blue) and linear fit of the maximum spore germination rate for each of the L- and D-ala concentration pairs measured. (**B**) Standard error of the mean for the measured germination rate shown in Fig. 2F. Errors come from n≥2 independent experiments with N≥250 spores in each experiment. (**C**) Experimentally measured germination rate as a function of an adjusted ratio of L- to D-ala concentration. D-ala$_0$=0.005 mM is the concentration of D-ala at which the germination response becomes ratiometric.

Figure S3

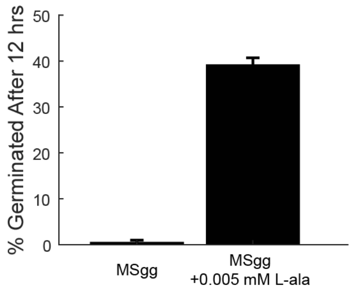

Figure S3. Germination on minimal media used for CFS production, related to Figure 3. Percent of germinated spores after 12 hours on agarose pads made from the minimal media MSgg. MSgg pads with the addition of 0.005 mM L-ala is shown for comparison with a very low concentration of germinant. Error bars represent the mean ± SEM of N>200 spores.

Figure S4

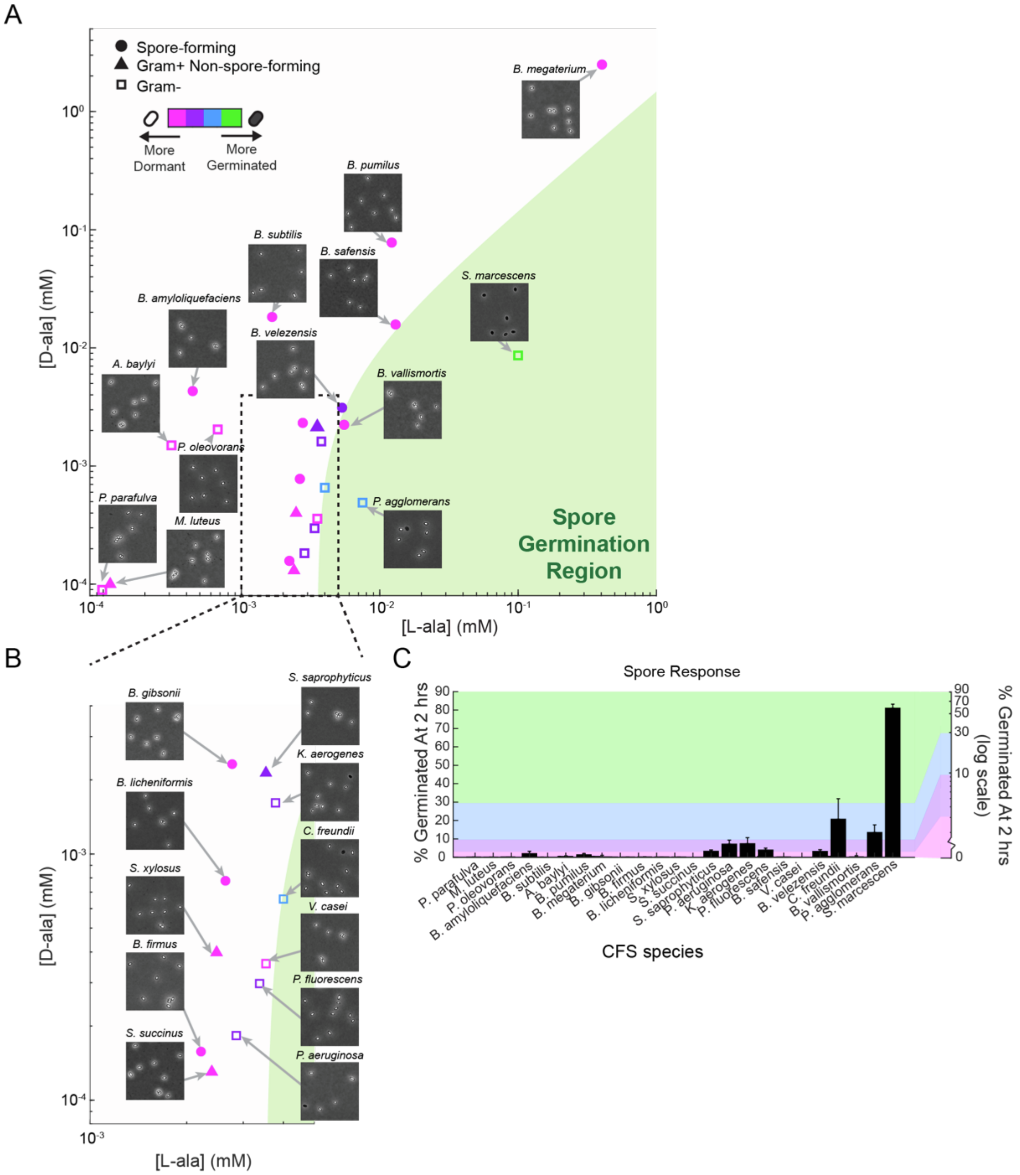

Figure S4. Germination response of *B. subtilis* spores to different species CFS, related to Figures 3 and 4. (**A**) Representative phase contrast images of *B. subtilis* spores on CFS from each bacterial species. As in Fig. 3C, each species marker is placed using the L- and D-ala concentration measurements from LC-MS with (●) spore forming, (▲) Gram+ non-spore-forming, and (□) Gram- species indicated. The region causing germination of *B. subtilis* spores (from the model in Fig. 2I) is indicated in green. The relative germination of *B. subtilis* spores on each species CFS after two hours is indicated using magenta-to-green color scale (as measured using phase-contrast microscopy). (**B**) Inset of region in (A) indicated by the dashed box. (**C**) Percent of *B. subtilis* spores germinated after 2 hour incubation on a pad of each species CFS. Species are organized in ascending order of germination rate as predicted from the analytical measurement of L- and D-ala concentration from each species CFS combined with the germination response model. The colored regions show the thresholds used to color code species response in Fig 4C and Fig. S4A-B. Data represent mean ± SEM from N>300 spores for each species.

Figure S5

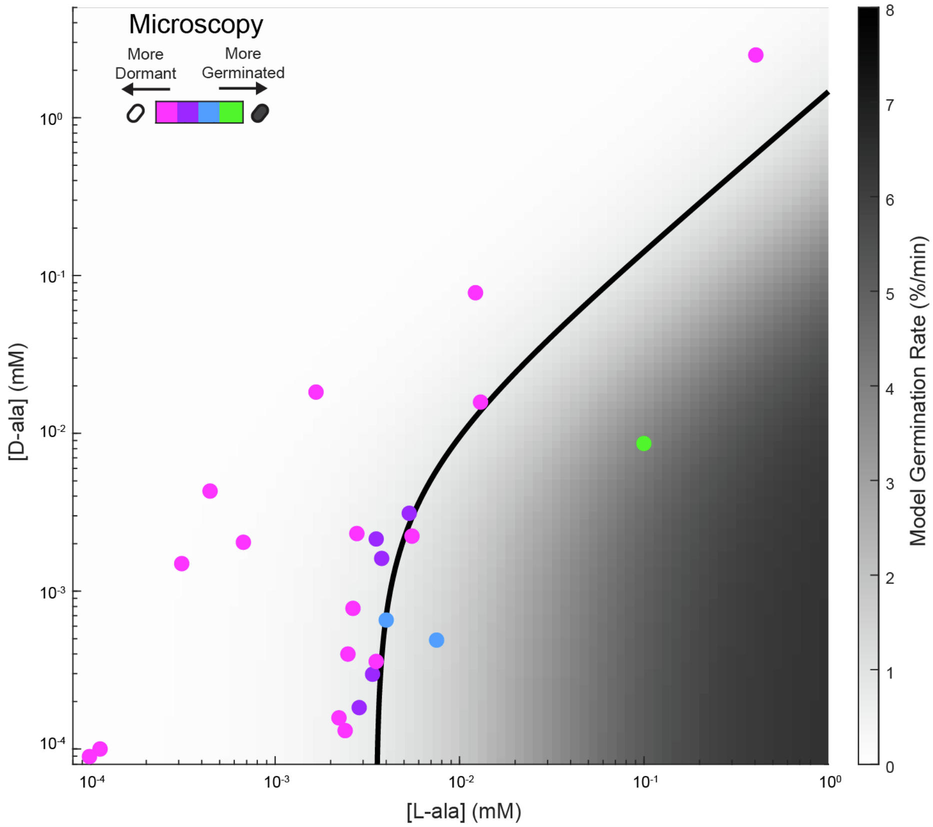

Figure S5. Germination Response Model and results from CFS from other bacterial species, related to Figures 3 and 4. As in Fig. 4C, the relative germination of *B. subtilis* spores on CFS from each species after two hours is indicated using magenta-to-green color scale (as measured using phase-contrast microscopy). Each species dot is placed using L- and D-ala concentration measurements from LC-MS, and the simulated germination response map is shown in gray scale (from the model in Fig. 2I). The black line shows the threshold used to define the region causing germination of *B. subtilis* spores shown in Fig. 3C, 4C and S4.

Figure S6

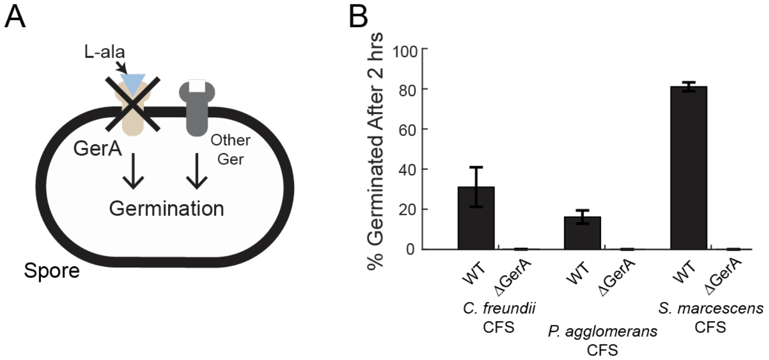

Figure S6. L-ala triggers germination of *B. subtilis* spores in CFS from select species, related to Figure 4. (**A**) Schematic of testing L-ala-induced spore germination by deleting the L-ala germinant receptor GerA. (**B**) Percent of wild type (WT) or Δ*gerA B. subtilis* spores germinated after 2 hour incubation on a pad of each species CFS. Data represent mean ± SEM.

Figure S7

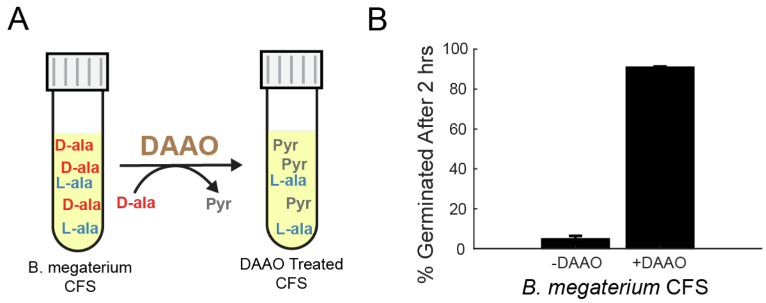

Figure S7. D-ala in CFS inhibits germination of *B. subtilis* spores, related to Figure 4. (**A**) Schematic showing how D-amino acid oxidase (DAAO) catalyzes the deamination of D-ala to pyruvate. (**B**) Percent of germinated *B. subtilis* spores after 2 hours incubation on pads made from *B. megaterium* CFS and *B. megaterium* CFS treated with DAAO. Data represent mean ± SEM.

Figure S8

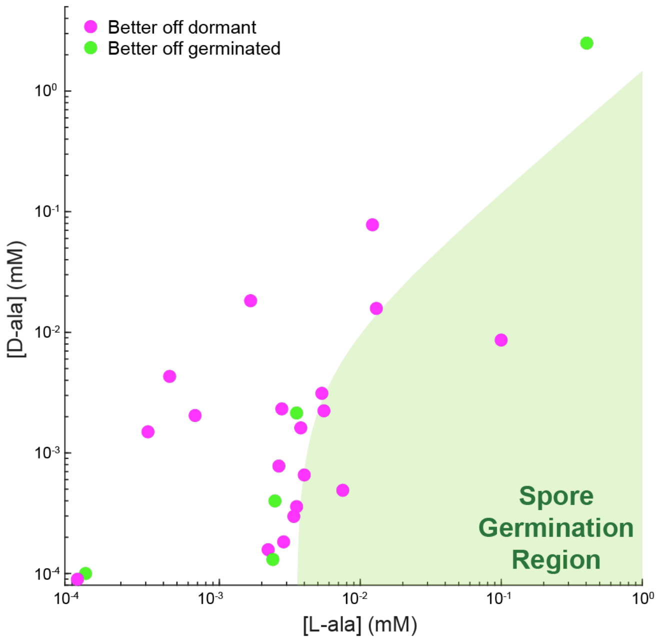

Figure S8. Cell-spore competition results versus native spore germination, related to Figure 5. (**A**) Competition assay results for each species are shown with magenta for "Better Dormant" and green for "Better Germinated." As in Fig. 3C, each species marker is placed using the L- and D-ala concentration measurements from LC-MS and the region causing germination of *B. subtilis* spores (from the model in Fig. 2I) is indicated in green.

Table S1.

Model parameters, related to Figure 2. Table of parameters used in the equilibrium binding kinetics model. See methods for details of model.

**Parameters**	**Description**	**Value - This work**	**Value - Ref 26**	**Units**
k_l	L-ala dissociation constant	0.026	0.012	mM^{-1}
k_d	D-ala dissociation constant	0.0052	0.0076	mM^{-1}
α	Maximum germination rate	6.6	3.6	%/min

Table S2.

Bacterial species, related to STAR Methods. Table of the bacterial species used in this study. Growth OD is their $OD_{600\ nm}$ after 24 hours growth in MSgg. Strain Sources: [1]University of Maryland, College Park, MD, Ref. 39 [2]University of California at San Diego, La Jolla, CA [3]Previously University of California at San Diego, La Jolla, CA

Species	OD After 24 hrs	Gram Staining	Spore Forming	Growth Temp. (°C)	Source
Bacillus subtilis	2.9	+	+	37	Wade Winkler laboratory[1]
Bacillus licheniformis	2.5	+	+	37	Kit Pogliano laboratory[2]
Bacillus amyloliquefaciens	2.3	+	+	37	Kit Pogliano laboratory[2]
Bacillus gibsonii	3	+	+	37	Provided by AGBiome
Bacillus vallismortis	2.7	+	+	37	Provided by AGBiome
Bacillus velezensis	2.6	+	+	37	Provided by AGBiome
Bacillus firmus	2.8	+	+	37	Kit Pogliano laboratory[2]
Bacillus megaterium	2.9	+	+	37	Kit Pogliano laboratory[2]
Bacillus safensis	2.8	+	+	37	Provided by AGBiome
Bacillus pumilus	2.8	+	+	37	Kit Pogliano laboratory[2]
Micrococcus luteus	1.9	Variable	-	37	Kit Pogliano laboratory[2]
Staphylococcus xylosus	1	+	-	37	Rachel Dutton laboratoy[3]
Staphylococcus saprophyticus	1.4	+	-	30	Rachel Dutton laboratoy[3]
Staphylococcus succinus	1.2	+	-	37	Rachel Dutton laboratoy[3]
Pseudomonas aeruginosa	1.4	-	-	37	Suel Lab library
Klebsiella aerogenes	2.9	-	-	37	Suel Lab library
Pseudomonas fluorescens	1.7	-	-	30	Suel Lab library
Citrobacter freundii	2.7	-	-	37	Suel Lab library
Serratia marcescens	2.8	-	-	37	Suel Lab library
Pantoea agglomerans	1.8	-	-	37	Kit Pogliano laboratory[2]
Vibrio casei	2.9	-	-	30	Rachel Dutton laboratoy[3]
Pseudomonas parafulva	2.9	-	-	30	Kit Pogliano laboratory[2]
Pseudomonas oleovorans	2	-	-	37	Jeff Hasty laboratory[2]
Acinetobacter baylyi	2.6	-	-	37	Jeff Hasty laboratory[2]

Table S3.

Alanine enantiomer composition of CFS, related to Figure 3. The concentrations of L- and D-alanine in each species CFS as measured by LC-MS/MS.

**Species**	**L-alanine (mM)**	**D-alanine (mM)**
Bacillus subtilis	0.00166	0.01827
Bacillus licheniformis	0.00264	0.00078
Bacillus amyloliquefaciens	0.00044	0.00431
Bacillus gibsonii	0.00277	0.00232
Bacillus vallismortis	0.00552	0.00223
Bacillus velezensis	0.00534	0.00312
Bacillus firmus	0.00222	0.00016
Bacillus megaterium	0.40437	2.49721
Bacillus safensis	0.01300	0.01577
Bacillus pumilus	0.01220	0.07784
Micrococcus luteus	0.00011	ND
Staphylococcus xylosus	0.00248	0.00040
Staphylococcus saprophyticus	0.00353	0.00214
Staphylococcus succinus	0.00240	0.00013
Pseudomonas aeruginosa	0.00286	0.00018
Klebsiella aerogenes	0.00378	0.00161
Pseudomonas fluorescens	0.00337	0.00030
Citrobacter freundii	0.00400	0.00066
Serratia marcescens	0.09991	0.00863
Pantoea agglomerans	0.00751	0.00049
Vibrio casei	0.00353	0.00036
Pseudomonas parafulva	0.00010	0.00009
Pseudomonas oleovorans	0.00031	0.00150
Acinetobacter baylyi	0.00067	0.00204
